# Abstracts from the International Science Symposium on HIV and Infectious Diseases (ISSHID 2019): Proceedings: Chennai, India. 12-14 October 2019

**DOI:** 10.1186/s12919-020-00190-4

**Published:** 2020-06-19

## ISSHID Abstract-107 Effects of marine extracts on augmenting cytokine production and its anti tumor activity

### Muthuraman Muthuchamy^1^, Manikannan Mathaiyan^1^, Krupakar Parthasarathy^1^, Lavanya Babu^1^, Karthikeyan M^2^, Ashok G^2^ , Sivasankaran Munusamy Ponnan^3^, Luke Elizabeth Hanna^3^

#### ^1^Center for Drug Discovery and Development, Sathyabama Institute of Science and Technology, Chennai, India; ^2^Department of Microbiology, Faculty of Medicine, Quest International University Perak, Ipoh, Malaysia; ^3^Department of HIV/AIDS, National Institute of Research in Tuberculosis, ICMR, Chennai, India

**Background:** Cytokines are pivotal in governing the immune responses by communicating to various immune cells and play vital role in cancer immunotherapy. Among all the cytokines IL-2, IL-21, IL-16, IFN-γ and TNF- α are key players in governing the antitumor T cells immunity. With this backdrop our study is conducted to investigate the anti tumor specific cytokine stimulation potential of selective marine extracts on human PBMCs.

**Methods:** Marine algal (Sargassum species) crude extracts and compounds were prepared by standard extractions procedure and phytochemicals analyzed by TLC and GCMS. Human PBMCs were isolated by using Histopaque (sigma) were treated with algal crude extracts and purified compounds (C1, C2, C3).After 12 hours of post stimulation stained with Th1/Th2/Th17/Th21 multiplex cytokine bead array kit (CBA). Percentage of cytokines stimulation levels were analyzed by multicolour flowcytometry.

**Results:** Of the several cytokines screened, IL-2 and IL-21 were stimulated significantly by aqueous extracts and compound 1 and compound 2. Though other cytokines were stimulated the levels were not significant when compare with control groups. Cytotoxicity of these extracts had done by MTT assay and none of the extracts have shown toxicity up to 10 mg/ml.

**Conclusion:** These studies have shown the potential of IL-21 cytokine stimulation of marine algal extracts on human PBMCs. IL-21 is a potent stimulator or T cell antitumor Immunity. Structural identification of cytokine stimulating principle compounds are under process.

## ISSHID Abstract-266 Role of MYBPC3Δ25bp mutation in rheumatic heart disease among South Indian population

### Maheshkumar Poomarimuthu^1,2^, Sony Kadiam^1^, Saranya Devaraj^3^, Sankar Natesan^3^, Sivakumar Elango^4^, Jayalakshmi Mariakuttikan^1^

#### ^1^Department of Immunology, Madurai Kamaraj University, Madurai, Tamil Nadu, India; ^2^Multidisciplinary Research Unit, Madurai Medical College, Madurai, Tamil Nadu, India; ^3^Department of Genetic Engineering, Madurai Kamaraj University, Madurai, TamilNadu, India; ^4^Institute of Child Health and Research Centre, Government Rajaji Hospital, Madurai, Tamil Nadu, India

**Background:** Rheumatic heart disease (RHD) is an autoimmune disease caused by exaggerated host immune response to group A Streptococcal infection via molecular mimicry. MYBPC3, which encodes myosin-binding protein C, cardiac-type (MYPC3) is vital for the structural maintenance and regulation of the heart muscles. A 25 bp deletion of MYBPC3, has been associated with an increased risk of various cardiovascular diseases. Hence, the present study intended to investigate whether the MYBPC3Δ25bp influence the development and severity of RHD.

**Methods:** The study includes 90 RHD patients (68 mitral valvular lesions (MVL) and 22 combined valvular lesions (CVL) patients) and 74 healthy siblings enrolled at Government Rajaji Hospital, Madurai with prior informed consent and ethical clearance. The presence of MYBPC3Δ25bp was determined by PCR method. Pairwise multiple sequence alignment was also performed with PepM5 protein of Streptoccocus and MYPC3 using EMBOSS.

**Results:** The frequency of heterozygous MYBPC3Δ25bp was significantly decreased in RHD patients (OR=0.25; p=0.03) and wild homozygous was significantly high in RHD patients (OR=4.01; p=0.03) when compared to healthy siblings. While, homozygous MYBPC3Δ25bp was completely absent in both RHD patients and healthy siblings. The MYPC3 exhibited 24.4% sequence identity with the pepM5 protein which is similar to that of myosin (25.2%) and tropomyosin (25.7%).

**Conclusion:** The present study suggests that MYPC3 protein may exhibit molecular mimicry with the Streptococcal M protein and heterozygous MYBPC3Δ25bp might be associated with protection towards RHD in South Indian population. However, further experimental validation is warranted to substantiate the present findings.

## ISSHID Abstract-403 Differences in susceptibility of primary and cancer cells to Chandipura virus

### Reshma Koolaparambil Mukesh^1,2^, John B Johnson^1^

#### ^1^Pathogen Biology, Virology, Rajiv Gandhi Centre for Biotechnology, Trivandrum, Kerala, India; ^2^Manipal University, Manipal, Karnataka, India

**Background:** Oncolytic virotherapy is an emerging alternative approach to target cancers employing cytopathic viruses. The oncolytic potential of Chandipura Virus (CHPV) , A human rhabdovirus, has never been investigated into. Here we explore the possibility of exploiting the cytolytic potential and interferon ( IFN) susceptibility of CHPV to target cancers.

**Methods:** Mammalian cells namely A549, U138, PC3, Hep G2, HeLa and Human Adult Dermal Fibroblast (HADF) were infected with CHPV and the cytopathic effects were assesses using a phase-contrast microscope at regular intervals for 48 h. Cell viability and growth kinetics were determined by MTT and plaque assays respectively. To assess the IFN mediated protection, cells were infected with 0.1 MOI of 24 h post IFNβ pre-treatment (1000U/ml) and the cell viability were estimated.

**Results:** Cancer cells exhibited marked cytopathic properties compared to primary cells upon CHPV treatment. Cell viability was markedly reduced with 0.1 MOI of CHPV at 48hpi in all the cells excluding HADF. Delayed viral growth kinetics was observed in HADF. Cancer cells were highly susceptible to CHPV yielding maximum viral titre as early as 24 hours which was delayed in HADF by 48 hours. Cell viability and viral titre were significantly reduced upon IFN treatment in all the cells except U138.

**Conclusion:** CHPV is a promising oncolytic vector with enhanced cytopathicity in cancer cells. The increased susceptibility of U138, a glioblastoma cell line, to CHPV even after the pre-treatment with IFN, suggests that with further attenuations and modifications, CHPV can be used against glioblastoma.

## ISSHID Abstract-67 T lymphocyte Negative regulatory molecule CTLA-4 +49A/G polymorphism in Rheumatic Heart Disease patients among South Indians

### Sony Kadiam^1^, Maheshkumar Poomarimuthu^1,2^, Sivakumar Elango^3^, Jayalakshmi Mariakuttikan^1^

#### ^1^Department of Immunology, Madurai Kamaraj University, Madurai, Tamil Nadu, India; ^2^Multidisciplinary Research Unit, Madurai Medical College, Madurai, Tamil Nadu, India; ^3^Institute of Child Health and Research Centre, Government Rajaji Hospital, Madurai, Tamil Nadu, India

**Background:** Rheumatic heart disease (RHD) develops as a sequel to rheumatic fever (RF) manifested by group A Streptococcal infection in a susceptible host. Molecular mimicry and autoreactive T-lymphocytes play a decisive role in the pathogenesis of RHD. Cytotoxic T-lymphocyte antigen-4 (CTLA-4) is a major negative regulator of T cell activation. Hence, the present study aimed to identify whether the functional genetic polymorphism of CTLA-4 influences the severity of RHD.

**Methods:** Ninety nine patients (RF, N=9; RHD, N=90) were recruited from the Govt. Rajaji Hospital, Madurai. Based on the extent of valvular lesions, the RHD patients are clinically classified as patients with mitral valvular lesions (MVL, N=68) and combined valvular lesions (CVL, N=22). Blood samples (3-5ml) were collected from all participating individuals with due consent and prior ethical clearance. Genomic DNA was extracted by salting-out method. The CTLA-4 +49A/G polymorphism was genotyped by tetra-primer amplification refractory mutation system–polymerase chain reaction (T-ARMS-PCR) method. Statistical evaluation was performed using Epi-info v7.

**Results:** The frequency of CTLA-4 +49A allele was reduced in MVL (60% vs. 84%, OR=0.29, p=0.07) and CVL (75% vs. 84%, OR=0.6, p=0.7) when compared to RF patients. Correspondingly, the AA genotype frequency was also low among MVL (37% vs. 67%, OR=0.29, p=0.1) and CVL (55% vs. 67%, OR=0.6, p=0.7) than RF patients. However, there was lack of statistical significance.

**Conclusion:** As RHD is a complex disease, several genetic factors might contribute to its pathogenesis, including CTLA-4. Further, larger studies are warranted to substantiate this finding.

## ISSHID Abstract-342 Predictive Value of Neck Circumference to Thyromental Distance Ratio for Difficult Intubation in Obese Patients

### Nivedha Senkuttuvan Pillai, R Archana, Ratna Paramaswamy

#### Saveetha Medical College, Thandalam, Chennai, India

**Background:** Many predictors exist for difficult intubation but lack diagnostic accuracy in obese individuals. This study is an attempt to analyze the use of neck circumference to thyromental distance ratio as a new predictor for the possible difficulty in intubation.The objectives are to compare the difficulty level of intubation between the obese and non-obese patients using intubation difficulty scale and to evaluate the efficacy of neck circumference to thyromental distance ratio as an effective predictor for difficult intubation in obese patients.

**Methods:** The study was conducted after obtaining ethical committee approval and informed consent from the patients. This was a prospective observational study where 150 patients comprising of both obese and non- obese categories were selected, and their body mass index, neck circumference, thyromental distance were measured and analyzed using unpaired student’s t test and logistic regression tests.

**Results:** Among obese and non-obese patients, body mass index, neck circumference, ratio of neck circumference and thyromental distance, Cormack Lehane grade and intubation difficulty scale were found to be significantly higher in obese patients which indicated a significantly higher difficulty level of intubation. Positive correlation was observed between ratio of neck circumference to thyromental distance with Cormack Lehane grade and Intubation difficulty scale, which was statistically significant (P<0.0001).

**Conclusion:** In conclusion, difficult intubations were more common in the obese patients was strongly associated with the increase in the NC/TMD ratio and a preoperative value of NC/TMD≥4.0 was considered to be a good predictor of difficult intubation in obese patients.

## ISSHID Abstract-351 Effect of reflexology on milk secretion among postnatal mothers who had undergone caesarean section

### R.Archana, D.PadmaPriya, S.Kalabharathi

#### Saveetha College of Nursing, Saveetha Institute of Technical and Medical Sciences (SIMATS), Thandalam, Chennai, India

**Background:** Reflexology, an ageold natural therapy, the foot reflex points are stimulated when pressure is given along the areas of foot using fingers and thumbs which increase the amount of hormones secreted by anterior pituitary and enhances lactation.

**Method:** Quasi experimental study was conducted among 60 caesarean mothers,30 in experimental and 30 in control group , after obtaining ethical committee approval and written informed consent . Pretest was done using the breast pump to assess the level of milk secretion in both the groups and the experimental group was given the reflexology. Post test was assessed in both the groups on the 4th day using the breast pump.

**Results**: In control group, the level of milk secretion was inadequate in 28 (93.33%) and moderate in 2 (6.67%) . On the 4th day with no intervention, the posttest assessment revealed that the level of milk secretion was moderate in 6 (20%) and inadequate in 24 (80%) where as in experimental group, the level of milk secretion was inadequate in 26(86.67%) and moderate in 4 (13.33%) before the intervention. After administering reflexology the posttest assessment revealed that the level of milk secretion was adequate in 25 (83.33%) and moderate in 5 (16.67%). The improvement mean in experimental group was 7.63 with SD 1.07 and t value 19.430 which was highly significant at p< 0.001

**Conclusion:** Milk secretion among caesarean mothers was enhanced after administering reflexology. Therefore it can be used as successful nursing intervention to caesarean mothers for promoting lactation

## ISSHID Abstract-361

**Withdrawn.**

## ISSHID Abstract-L12 Comparison of safety & efficacy of Metformin plus Glimepiride vs Metformin plus Sitagliptin in patients of Type 2 Diabetes mellitus with poor glycemic control, a randomized open label study

### Srinivasan .V

#### Department of Pharmacology, Saveetha Medical College & Hospital, Saveetha Institute of Medical & Technical Sciences (SIMATS), Chennai, India

**Background:** The highest number of people with diabetes in the world will be in India in near future. The IDF has subsequently released estimates of the numbers of people with diabetes for 2003 and forecasts for 2025 of 194 million and 334 million. Incretin-based therapies, principally DPP-4 inhibitors, have slowly gained traction in the therapy of type 2 diabetes. There is not much comparative drug study comparing fixed dose combination of Metformin plus Glimepiride Vs Metformin plus Sitagliptin in patients of type 2 diabetes mellitus with poor glycemic control.

**Methods:** The study duration was 12 weeks, each randomized patient in two groups were assigned study drugs and were assessed periodically.

**Results:** Out of the 74 patients screened, 60 patients who satisfied the eligibility criteria were randomized into two treatment groups namely the Metformin plus Glimepiride and the Metformin plus Sitagliptin, consisting of 30 patients and 30 patients respectively. Among 60 patients randomized, 57 completed the study till 12 weeks study duration while 3 patients discontinued the study.

**Conclusion:** From our study it showed that combination drug of sitagliptin with metformin is non inferior as the other combination drug of Glimepiride with metformin. Moreover the sitagliptin and metformin combination is associated with fewer incidences of adverse events when compared with Glimepiride and metformin.

## ISSHID Abstract-230 In vitro anticandidal activity of Hedyotis corymbosa

### Samudhra K^1^, Suganthi P^1^, Swathi M^1^, Aneesha S^1^, Selvam P^2^

#### ^1^Department of Microbiology, Dr. ALM Post Graduate Institute of Basic Medical Sciences, University of Madras, Taramani, Chennai-600113, India; ^2^Aravind Herbal Lab, Rajapalayam-626117, India

**Background:** Candidiasis is a serious health infection affecting various parts of the body, especially the immunocompromised. Because of emerging resistance to the antifungal drugs and their side effects, effective and secure therapy is needed. Plant compounds are generally known for their medicinal properties including antimicrobial activity. In this study anticandidal activity of Hedyotis corymbosa extract against urinary tract and vaginal candidiasis was studied.

**Methods:** A total of 60 Candida isolates were included in this study, 17 from diabetic urine and 43 from vaginal swabs. Candida species were identified by standard protocol. Antifungal susceptibility test was done by disc diffusion method as per CLSI guidelines. The aqueous plant extract was obtained and mixed with different solvents. The anticandidal activity of the extract was tested by well diffusion method. Minimum inhibitory concentration (MIC) was calculated by microdilution method and the minimum fungicidal concentration (MFC) was noted.

**Results:** Among the 60 isolates, itraconazole and fluconazole showed 70.37% and 64.15% resistance respectively, and majority of the isolates were sensitive to the plant extract. The extract showed anticandidal activity with MIC 50-25μg/ml for the Candida isolates.

**Conclusion:** The anticandidal activity of Hedyotis corymbosa was determined and it was found to be potent for the clinical isolates which were resistant to antifungal drugs.

## ISSHID Abstract-184 Efficient inhibition of HIV by the aqueous crude extracts of Artabotrys odoratissimus an Indian medicinal plant

### Kalaivani.V, Devi.G, Deepika. L, Elanchezhiyan Manickan

#### Department of Medical Microbiology, Dr.ALM PGIBMS, University of Madras, Taramani Campus, Chennai, India

**Background:** Human Immunodeficiency Virus (HIV) causes Acquired Immunedeficiency Syndrome (AIDS) is one of the most serious health challenges over the world. They are many antiretroviral drugs for the HIV, but it has been found to have some side effects. Therapeutic properties of herbal medicine have been receiving a highlightened interest in the entire health care horizon. *Artabotrys odarastissimus* a member of family Annonaceae is widely distributed in Asia and the southern part of China is known for its medicinal bioactivities which include Antidepressant, Mood elevator, Antiseborrhoeic, Antiseptic, and contraceptive properties. The current work is aimed to evaluate the anti-HIV activity of *Artabotrys odarastissimus*.

**Methods:** Aqueous crude extract of flower and leaf of *Artabotrys odarastissimus* is taken and evaluated for anti-HIV activity by HIV gag P24 inhibition assay on human PBMC. The toxicity of the extract was measured by MTT assay using vero cells.

**Results:** We found that the Aqueous crude extract of *Artabotrys odarastissimus* inhibited the replication of HIV as revealed by the absence of HIV gag p24 core protein. The percentage of inhibition of *Artabotrys odarastissimus* is 96.4% at 1.5pg/ml viral concentration at 50μg/ml of plant extract. Besides it was found to be that the aqueous crude extract was non-toxic on the vero cells. It shows 80-90% of cell viability at 45μg/ml of extract

**Conclusion:** Our study revealed the medical utility of *Artabotrys odarastissimus*. Besides it non-toxic nature at the tested concentrations. Further studies may reveal more of their medical property in detail.

## ISSHID Abstract-410 Antiviral activity and Molecular docking studies of Enicostemmaaxillare-leaves against HIV

### Anbalagan S, Sankareswaran M, Moorthy M

#### Department of Microbiology Department, Muthayammal College of Arts and Science, Rasipuram, Namakkal, TamilNadu, India

**Background:** Current research in drug discovery from medicinal plants involves a multifaceted approach combining botanical, phytochemical, biological, and molecular techniques. Medicinal plants drug discovery continues to provide new and important leads against various pharmacological targets including Cancer, HIV/AIDS, Alzheimer’s, Malaria, etc,. Several natural products drugs of plant origin have either recently been introduced to the United States markets, including art ether, galatamine, nitisinone, and tiotropium or currently involved in late phase clinical trials. Realising the importance of HIV/AIDS, knowing the immense value of Indian medicine plants and attempt was made to study the anti viral properties of these plants, to investigate the antiviral, phytochemical screening and molecular docking of *Enicostemma axillare*-Leaves against HIV.

**Methods:** The phytochemical analysis, antiviral, GC-MS analysis and molecular docking studies Molecular docking studies of Enicostemma axillare-Leaves against HIV.

**Results:** The phytochemical study was done, the anti-HIV property of the plants was evaluated based on its ability to inhibit the HIV reverse transcriptase enzyme and several plants have shown potential HIV inhibitory activity in-vitro. The high intensity signals obtained by GC-MS against methanolic extract of Enicostemma axillare contain fifteen spectrum of compounds. The compound 2-chloro ethyl linoleate was docked with HIV receptor (1W5V) and the dock score was 75.567.

**Conclusion:** From the results obtained, it could be concluded that the herb exhibited excellent antiviral activity.

## ISSHID Abstract-64

**Withdrawn.**

## ISSHID Abstract-79 Antimicrobial activity of selected in Indian medicinal plants

### P. Selvam^1^, P. Suganthi^2^, M. Anandhi^2^

#### ^1^Aravindh Herbal Labs (P) Ltd, Rajapalayam, Tamilnadu; ^2^Dept. of Microbiology, University of Madras-Taramani Campus, Chennai

**Background:** Medicinal plants are the excellent source for discovery of potential antimicrobial agents and chemical constituents present in the natural products provide the lead for the developments of effective drugs against bacteria and fungus. Extracts of polyherbal formulations used for developments of effective and board-spectrum antimicrobial agents. Present work is to investigate the anti-microbial activity of ethanol extract of selected some medicinal plants for identification of potential antimicrobial agent

**Methods:** Ethanol extract prepared from Scopariadulcis, Acacia ferruginea and Oldenlandiaumbellata by using hot continues extraction method and extract dried under vacuum. Dried extract is tested for panel of human pathogenic bacteria such as *E coli, S.auerus, Klebsiella pneumoniae Pseudomonas aeruginosa, Enterococcus, B.cerus,* and *Candida albicans* by disc diffusion method in nutrient agar medium

**Results:** All extracts had inhibitory activity against *E coli, S. auerus, Pseudomonas aeruginosa, K. pneumoniae* and *Candida albicans* and inactive against *Bacillus cerus.* Extract significant activity against *Klebsiella pneumonia, Pseudomonas aeruginosa* and *Candida albicans*.

**Conclusion:** Ethanol extract of above tested medicinal plants good source for searching of potential bioactive molecules with antimicrobial activity and Suitable for further studies to understand antimicrobial mechanism and for isolation of active compounds.

## ISSHID Abstract-97

**Withdrawn.**

## ISSHID Abstract-212 Repurposing of Nano-Herbomineral Formulation for Anti-HIV Activity

### Malarvizhi K^1^, Fang H^2^, Luo RH^2^, Zheng YT^2^, Vedha Hari BN^1^, Ramyadevi D^1^

#### ^1^School of Chemical & Biotechnology, SASTRA Deemed University, Thanjavur, Tamil Nadu, India; ^2^Kunming Institute of Zoology, Chinese Academy of Sciences, Kunming, Yunnan, China

**Background:** The classical Siddha medicine has an extensive role against chronic diseases, including the treatment of HIV/AIDS with the combination of formulations like Rasagandhi Mezhugu, Amukkara Chooranam, Nellikkai Lehyam (RAN). Sivanar Amirtham is one such higher-order medicine with eclectic activity against vatha (80), pitha (40), kapha (20) and 5 different respiratory diseases and also as an antidote for insect poison. The objective of the present work is to repurpose this medicine for anti-HIV activity and explore its characteristics and cytotoxicity.

**Methods:** Sivanar Amirtham the herbomineral Siddha formulation was characterized for its physicochemical properties such as particle size, FTIR, XRF, and TGA-DSC. The cytotoxicity and therapeutic anti-HIV activity of the sample were studied by MTT assay in C8166 cell lines and Syncytium formation inhibition assay using HIV-1IIIB strain infected cells, correspondingly.

**Results:** The average particle size was found to be 337.5 nm with a polydispersity index of 0.53. The FTIR and XRF analysis displayed characteristic functional groups of the active ingredients and presence of elements. TG-DSC showed an endothermic peak at 93.90°C and the linear weight loss from 95% to <20% in the temperature range of 121°C-486°C. The nano-herbomineral formulation revealed negligible cytotoxicity and anti-HIV activity at higher concentrations (EC50 >200 μg/mL) through MTT assay and syncytium inhibition assay, respectively.

**Conclusion:** Sivanar Amirtham is non-toxic and effective against HIV with various bioactive compounds, therefore repurposing the herbomineral formulation could provide an option in anti-HIV therapy for the prevention of sequela.

## ISSHID Abstract-222 Antimicrobial activity of *Chondrococcus hornemanni* against drug resistant vaginal isolates

### Swathi M^1^, P.Sugathi^2^, Latha V^3^, M Anandhi^4^, Aneesha S

#### Department Of Microbiology, University Of Madras, Dr ALMPGIBMS Taramani Chennai, India

**Background:** Vaginal discharge is a common clinical problems among women of reproductive age groups with multiple etiologies. It is the second most common problems after menstrual disorders. A complex of microorganisms maintains the normal vagina flora. In a developing country, where antibiotics are easily purchased without prescription from pharmaceutical and chemical stores so this kind of improper use of antibiotics will leads to development of drug resistance . Most of the drug resistant exhibit co-resistance to many other classes of antibiotics resulting in limitation of therapeutic options. Seaweeds are considered as an alternative source of bioactive compound with antibacterial and antifungal activity .

**Methods:** A total of 40 vaginal isolates were collected and identified according through standard procedure. Antimicrobial sensitivity test profiling of the isolates were carried out by adopting the Kirby-bauer disc diffusion method. Antimicrobial activity of crude extract of *C.hornemanni* against drug resistance vaginal isolates done by disc diffusion method.

**Results:** Among 40 isolates17(43%) E.faecalis,11(28%) *K.pneumoniae*,8(20%) E.coli,4 (10%) *Pseudomonas* spp. out of 23 (58%) of gram negative bacilli 10(25%) ESBL,6(15% ) Fluroquinolones resistant and 5(13%) were Aminoglycoside resistant. *C.hornemanni* showed a broad and high antibacterial activity against vaginal isolates of 13(76% ) *E.faecalis* followed by 2(18%) of *K.pneumoniae.*

**Conclusion:** In the present study, methonal extract of (red algae ) *C.hornemanni* seaweed against *E.faecalis* and *Klebsiella pneumoniae* showed broad inhibitory activity than antibiotic . The antibacterial activity of this seaweed were shown higher activity in *E.faecalis* than *Klebsiella pneumoniae* .

## ISSHID Abstract-341 Screening of phytochemicals, evaluation of anti-tuberculosis and antioxidant activities of Diospyrosis melanoxylon Roxb. by in vitro approach

### Vijayan Priyadharshni^1^, Venkataswamy Roopa^1^, Souprayane Aroumougame^1^, Narayanasamy Mathivanan^1^, V.N.Dusthackeer^2^, Mahizhaveni B.^2^, Thiruvengadam kasi^1^

#### ^1^Biocontrol and Microbial Metabolites Lab, Centre for Advanced Studies in Botany, University of Madras, Guindy Campus, Chennai, India; ^2^Department of Bacteriology ICMR-National Institute for Research in Tuberculosis, Chetpet, Chennai, India

**Background:** Tuberculosis is a serious Public Health problem due to increased multidrug resistant, which is caused by Mycobacterium tuberculosis. Medicinal plants offer a hope for developing alternate medicines for tuberculosis treatment by providing greater effectiveness with less toxicity.

**Methods:** Two different extracts of fruit and leaves (ethyl acetate and methanol) was investigated for the presence of phytochemicals by both qualitative and quantitative, in vitro antioxidant activities (DPPH, Iron chelation, Ferric thiocyanate, TBA and Phosphomolybdenum), antibacterial assay was done by well diffusion method on human pathogens, followed by, antitubeculosis activity was assessed on H37RV and MDR isolates and LRP assay was done using Rifampicin and Isoniazid as control.

**Results:** The results exhibited that, ethyl acetate and methanol extracts of leaves possessed maximum phytochemicals than that of fruit extracts of D. melanoxylon. Similar results were obtained in antioxidant assays (IC50 value of 18.01 and 163.4 μg/mL in DPPH assay and 383.3, 441.0 μg/mL in FRAP, respectively. Remarkable inhibitory effect was obtained against MRSA also methanol extracts showed superior antituberculosis activity (50-250 μg/mL) against standard H37RV, but clinical isolate are resistant to Isoniazid (H) and Rifampicin(R). Likewise, leaf methanol extract was found to have 96% reduction in Relative Light Units (RLU) against M. tuberculosis.

**Conclusion:** D. melanoxylon leaves are rich in phyto and bio compounds which could be a potential source of natural antioxidants and antibacterial agent for the treatment of diseases relating to the studied pathogenic bacteria.

## ISSHID Abstract-372 Effect of Yoga Nidra and Om chanting on blood pressure in hypertensive subjects

### R. Archana^1^, K. Anjana^2^, J.K. Mukkadan^1^

#### ^1^Department of Physiology, Saveetha Medical College, Saveetha Institute of Technical and Medical Sciences (SIMATS), Thandalam, Chennai, Tamil Nadu, India; ^2^Department of Physiology, Little Flower Medical Research Center, Angamaly, Kerala

**Background:** Hypertension and its complications are one among the leading cause of death and other impairments all over the world. In this study the effect of relaxation procedures Yoga nidra and OM chanting on blood pressure was assessed in hypertensive subjects.

**Methods:** After getting ethical clearance and written informed consent, 30 subjects (15 male, 15 female) who full filled the inclusion and exclusion criteria were enrolled in the study. Subjects received regular sessions of 20 minute yoga nidra followed by 5 minutes of OM chanting (3 OM/minute) from a yoga professional for 30 days from 8 to 8.30 am. Demographic data was collected from all subjects. Pretest and posttest values of Blood pressure (American Heart Association guidelines), breath rate and frequency domain parameters of heart rate variability were assessed.

**Results:** The study findings were statistically analyzed using paired t test. After 30 days of practice, there was a significant reduction in Systolic blood pressure from 137.60±7.15 to 126.33±5.43 mmHg, breath rate from 14.20±.1.15 to 10.30±.596 / minute (p≤0.0001) and caused no significant change in diastolic blood pressure. Pulse rate decreased from 74.67± 2.65 to 71.77±2.20 /minute (p≤0.0001). In heart rate variability, the High frequency component (HF) increased from 47.5±3.12 to 57.2±3.5 nu, there was a decrease in Low frequency component (LF) from 51.4±3.57 to 38.6±2.73 nu, followed by decrease in LF/HF 1.1±0.32 to 0.7±0.08 (p≤0.05) indicating parasympathetic stimulation.

**Conclusion:** Yoga nidra along with OM chanting was beneficial in regulating systolic blood pressure, pulse rate and heart rate variability in hypertension.

